# A Personalized Approach to the Management of Congestion in Acute Heart Failure

**DOI:** 10.17925/HI.2023.17.2.2

**Published:** 2023-11-23

**Authors:** Gustavo R Moreira, Humberto Villacorta

**Affiliations:** Cardiology Division, Fluminense Federal University, Niterói, Rio de Janeiro State, Brazil

**Keywords:** Acute heart failure, congestion, diuretic, renal function, urinary sodium, treatment

## Abstract

Heart failure (HF) is the common final pathway of several conditions and is characterized by hyperactivation of numerous neurohumoral pathways. Cardiorenal interaction plays an essential role in the progression of the disease, and the use of diuretics is a cornerstone in the treatment of hypervolemic patients, especially in acute decompensated HF (ADHF). The management of congestion is complex and, to avoid misinterpretations and errors, one must understand the interface between the heart and the kidneys in ADHF. Congestion itself may impair renal function and must be treated aggressively. Transitory elevations in serum creatinine during decongestion is not associated with worse outcomes and diuretics should be maintained in patients with clear hypervolemia. Monitoring urinary sodium after diuretic administration seems to improve the response to diuretics as it allows for adjustments in doses and a personalized approach. Adequate assessment of volemia and the introduction and titration of guideline-directed medical therapy are mandatory before discharge. An early visit after discharge is highly recommended, to assess for residual congestion and thus avoid readmissions.

Heart failure (HF), a prevalent disease, is the common final pathway of several conditions, which result in the activation of numerous neurohumoral pathways. Cardiorenal interaction plays an essential role in the progression of the disease, and the use of diuretics is a cornerstone in the treatment of hypervolemic patients, especially in acute decompensated HF (ADHF).^1^ The management of congestion is complex and may potentially be harmful or, sometimes, misinterpreted by the physician. For example, hypotension and worsening renal function may occur during the decongestion process, which can pose challenges to the introduction or titration of guideline-directed medical therapy (GMDT). To avoid errors and misinterpretations during decongestion in patients with ADHF, one must understand the complex interaction between the heart and the kidneys in this setting. The aim of this review is to provide an update on the management of congestion in ADHF, with a focus on diuretic therapy and the cardiorenal interactions resulting from its use. In addition, we propose an individualized approach, based on precision medicine concepts.

## Pathophysiology of acute decompensated heart failure

HF is the common final pathway of several cardiac disorders and is associated with high morbidity and mortality.^1^ Its primary manifestation, effort intolerance, depends mainly on the increased filling pressures of the left ventricle (LV) and low cardiac output (CO), which is reflected by the increased pulmonary capillary wedge pressure (PCWP).^1^ In patients with chronic HF, the sympathetic nervous system (SNS) and the renin-angiotensin-aldosterone system (RAAS) are activated, causing vasoconstriction and reduced excretion of sodium and water. These neuroendocrine activations are compensatory mechanisms that aim to increase CO and blood pressure through increased peripheral vascular resistance (PVR) and increased volemia, due to water and sodium retention. In the long run, however, these neurohormonal activations are deleterious, since congestion itself is harmful for both the heart and the kidneys.^2^

The kidneys receive about 25% of the CO and constitute a circulatory mesh of low-resistance vasculature, with secretion and filtration properties, and the ability to modify the composition of body solutes, as well as the amount of water in the body.^1^ Therefore, they play an important role in the pathophysiology of HF. Decompensated HF patients present with worsening of their condition, with acute dyspnea or worsening New York Heart Association functional class, caused by hypervolemia or fluid redistribution. Currently, diuretics are mandatory for symptom relief and mortality reduction, since congestion increases the risk of events, even in patients with pulmonary congestion without obvious hypervolemia.^1^

The interaction between the kidneys and the heart is the subject of current interest, mainly due to the bilateral characteristic of their relationship. The presence of chronic kidney disease (CKD) causes cardiac dysfunction (hypertrophy and atherosclerosis) and, on the other hand, patients with HF may develop a decline in renal function, mainly due to congestion.^2^ The so-called cardiorenal syndrome is an entity that recognizes the reciprocal effects of these two systems and classifies patients into one of five categories, depending on the primary organ involved and on setting, whether chronic or acute.^3^ Type I cardiorenal syndrome refers to patients with acute heart disease causing acute renal dysfunction, which is the case in ADHF that evolves with creatinine elevations during the patient’s hospital stay.

The symptoms of dyspnoea occur with the increase in PCWP, which we call pulmonary congestion. For some physicians the term congestion refers to pulmonary congestion, and hypervolemia refers to the edemigenic syndrome. However, to make it clear, it is better to use the terms ‘pulmonary congestion’ and ‘systemic congestion’, (or hypervolemia) since not all pulmonary congestion reflects hypervolemia. The systemic congestion directly correlates with the pressure in the right atrium, and the pulmonary congestion correlates with the left atrium and left ventricle pressures, with implications in the haemodynamic assessment and therapy.^3^

**Figure 1:**
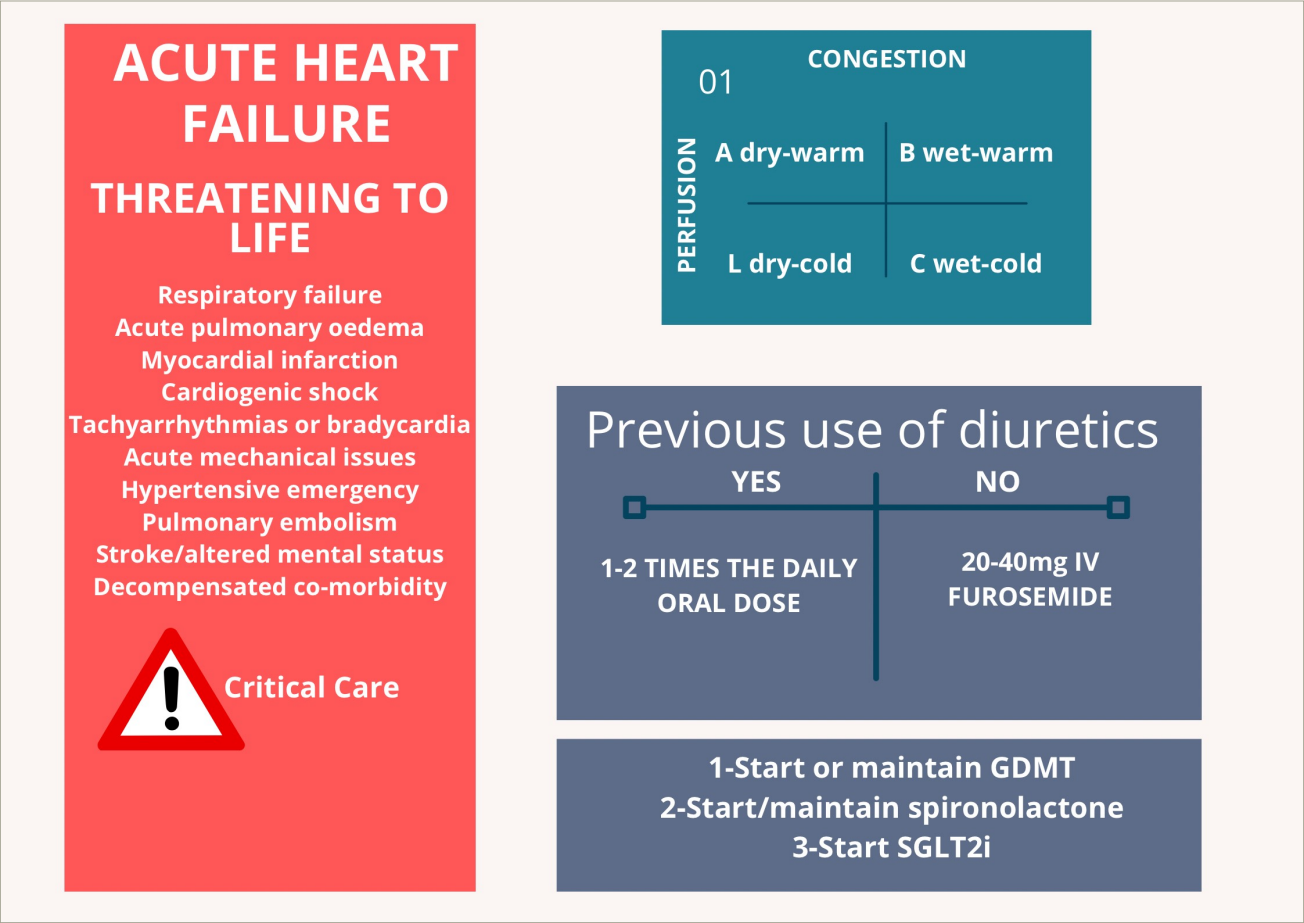
Initial evaluation and treatment of congestion in acute decompensated heart failure

To guide initial therapy in ADHF, guidelines recommend the determination of the haemodynamic profile based on clinical assessment. Stevenson proposed a simple classification depending on these haemodynamic profiles, in which the patient is classified into one of four categories, according to the assessment of peripheral perfusion and systemic congestion. These profiles are easily recognizable in their extremes, but the clinical accuracy both for diagnosing and for quantification of severity is poor in the presence of mild alterations.^4,5^ These haemodynamic profiles are demonstrated in *Figure 1*.

*Patients with life-threatening conditions should be identified and treated accordingly. The haemodynamic profile should be determined, to guide initial therapy. The initial dose of intravenous diuretic depends on the previous use of oral diuretic. GDMT, including spironolactone and SGLT2i, should be started or maintained unless specific contraindications exist.*

*GDMT = Guideline-directed medical therapy; SGLT2i = sodium glucose co-transporter 2 inhibitors.*

The dynamics of fluid exchange between the various compartments follow several physiological variables that are abnormal in the presence of HF and are influenced by pharmacotherapy. To better understand and correctly assess the volume status of patients with HF, some concepts of fluid dynamics are addressed below.

### Fluid compartments

The dynamics of fluids depend essentially on the differences in hydrostatic pressure or colloid-osmotic pressure between two compartments. The osmolarity and protein content of the plasma influence the fluid dynamics and pressure variations in the vessel wall, according to Laplace’s law.^3^ Both interstitial oedema and pulmonary oedema result from the extravasation of capillary fluid into the extracellular and alveolar spaces, respectively, due to an increase in hydrostatic pressures and/or a decrease in colloid-osmotic pressures. Of note, pulmonary congestion may occur without hypervolemia – a phenomenon known as fluid redistribution (from the splanchnic circulation to the lungs) – and, conversely, hypervolemic patients may present without pulmonary congestion.^3^

### Plasma refill rate

With increasing diuresis, the water is removed mainly from the intravascular space. This ‘emptying’ in the intravascular space is replaced by water taken from the interstitial space. This replacement is done at such a maximum rate, the so-called ‘plasma refill rate’.^6^ If the intravascular emptying rate is higher than the refill rate, transitory intravascular ‘hypovolemia’ may occur, causing transitory serum creatinine elevations. However, arterial hypotension is rarely observed and usually indicates true hypovolemia.^3,4^

### Diuretic efficiency

Diuretic efficiency is expressed as the volume of diuresis achieved per unit of furosemide measured in the urine.^3^ It is a measure of diuretic response, but it is challenging to perform in clinical practice.

### Diuretic resistance

Diuretic resistance is a term used to describe a poor response to diuretics resulting from pharmacokinetic, pharmacodynamic and functional changes in the nephron.^7^ Its prevalence increases with the duration of diuretic therapy and HF severity and is associated with worse prognosis.^8^ Although no standard definition exists, urinary output less than 100–150 mL/h or natriuresis less than 70 mEq/L after diuretic administration is suggestive of diuretic resistance.^3,4^ In clinical practice, diuretic resistance is identified by refractory oedema, despite high doses of diuretics.

### The braking phenomenon

This phenomenon results from morphological and functional changes that occur in the nephron when it is chronically exposed to loop diuretics. It is an adaptive response to a pharmacological intervention to avoid dehydration with the chronic use of diuretics. Increased capacity of sodium reabsorption in the distal portions of the nephron occurs in response to the increased loss of this solute. High tubular sodium concentrations due to diuretic therapy stimulates hyperplasia and hypertrophy of the distal portions of the nephron, leading to increased sodium reuptake, even after a single dose of diuretic.^7,9^ As a result, after the administration of loop diuretics in patients with HF, there is a significant increase in sodium exit from the proximal tubule and loop of Henle. However, little of this sodium actually reaches the urine (around 35%).^7^ The braking phenomenon explains, at least in part, the resistance to diuretics observed in some patients with HF.

## Initial assessment

The initial evaluation of patients with ADHF should include the identification of critical situations such as haemodynamic instability and/ or respiratory distress, which are not under the scope of this review. The haemodynamic profile should be determined to guide initial therapy.^1^ The vast majority of patients admitted to the hospital with decompensated HF are congested.^10^ In the BREATHE study, the Brazilian registry of ADHF, 85.2% of the patients had a wet and warm profile, indicating the presence of congestion with adequate peripheral perfusion.^7^ The patient should be asked about prior use of diuretics, since the approach is different depending on this information. Factors that may have precipitated the decompensation should be investigated. Previous GMDT should be maintained unless specific contraindications are present.^1^ The initial evaluation of ADHF is illustrated in *Figure 1*.

## Characterization of volemia and congestion

Patients with clear signs of hypervolemia, such as peripheral oedema, jugular vein distension, hepatomegalia and ascites, are easily identifiable. However, in some patients the determination of volemia remains a challenge even when supported by bedside ultrasound. The identification and quantification of congestion on clinical examination add prognostic value beyond symptoms and NT-proBNP levels and should not be neglected.^11^ The erroneous estimation of the degree of congestion, along with insufficient diuretic response, hydroelectrolytic abnormalities during the decongestion process, and misinterpretation of markers of renal function can lead to errors that may potentially be harmful. Likewise, determining when the patient has achieved the euvolemic state is a complex task, and some patients may not achieve optimal decongestion which has been associated with poor outcomes.^12,13^ Using bioelectrical impedance vector analysis (BIVA) our group along with three academic centers in Italy demonstrated that almost one third of the patients are discharged with residual congestion, including subclinical congestion. The identification of such patients is very important because they have worse outcomes.^14^

Isolated pulmonary congestion should be differentiated from true hypervolemia. Some patients present to the hospital with predominant pulmonary congestion and no signs of systemic congestion. This finding does not always mean hypervolemia. Fifty-four percent of patients admitted with acute HF had a weight gain ≤1 kg in the previous month, suggesting a more significant component of fluid redistribution.^15,16^ The European HF guidelines recommend this differentiation, to avoid excessive volume reduction in poorly distributed patients since this could worsen renal function.^1^ Fluid is moved from splanchnic circulation to the lungs due to venoconstriction but without hypervolemia. In this situation, treatment should be focused on vasodilators, rather than diuretics.

Physical examination is limited in assessing congestion in some cases. In a series of 50 patients with chronic HF, signs such as rales, oedema and jugular venous distension (JVD) were absent in 42% of patients with PCWP ≥22 mmHg.^17^ Chest radiography may be normal in 20% of congested patients,^11^ and bedside ultrasound has been suggested as a helpful tool in this scenario.^18^ An ongoing trial is assessing the role of inferior vena cava diameter evaluation, as well as vena cava respiratory variability for guiding diuretic therapy.^19^

The correct determination of congestion is crucial for safe discharge and to avoid readmissions.^13,20^ Clinical parameters and those derived from routine laboratory tests have insufficient sensitivity and specificity,^4^ and the European Society of Cardiology (ESC) recommends a multiparametric evaluation for assessing congestion in patients with HF.^1^ The qualitative and mainly quantitative evaluation of clinical or laboratory data, biomarkers, image tests and devices such as BIVA are useful tool to aid the decongestion process.^3^ NP-guided therapy in ADHF has failed to show benefits over conventional treatment.^21^ However, decreases of at least 30% in NP levels from admission to discharge has been associated with good prognosis.^22^ Absolute changes in haemoglobin during hospitalization has also been associated with haemoconcentration and, therefore, proper decongestion.^23^ In one study, absolute changes in haemoglobin from admission to day 7 was associated with lower 180 day mortality.^23^

With the portability of ultrasound equipment, bedside evaluation of pulmonary oedema, EF and inferior vena cava (IVC) variability reinforce the diagnostic arsenal, mainly in the acute HF setting. The measurement of pulmonary B-l ines demonstrated sensitivity of 94.1% (CI 81.3–8.3%) and the specificity is 92.4% (CI 84.2–96.4%) in a meta-analysis with 1,075 patients,^18^ and its use has been systematized for better reprodutibility.^24^ A diameter of IVC >21 mm has been associated with worsening renal function (WRF) and poor prognosis in both acute and chronic settings.^25,26^

**Figure 2:**
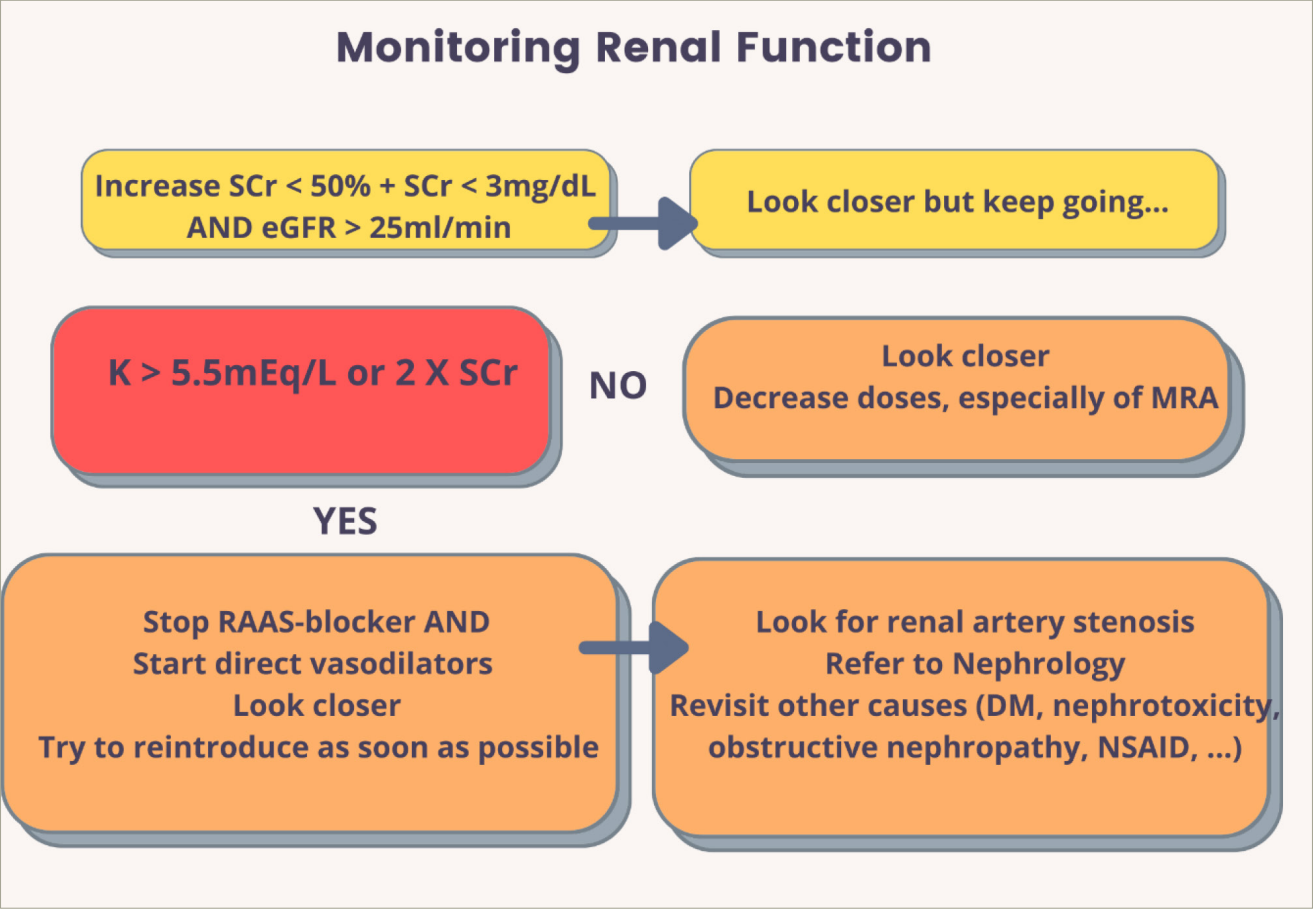
Monitoring renal function during the treatment of congestion in acute decompensated heart failure

*DM = diabetes mellitus; eGFR = estimated glomerular filtration rate; MRA = minelacorticoid receptor antagonists; NSAID = non-steroid anti-inflammatory drugs; RAAS = reninangiotensin-aldosterone system; sCR = serum creatinine.*

**Table 1:** Classification of acute kidney damage

	UO	Cr		
		KDIGO	AKIN	RIFLE
**Grade 1**	<0.5 ml/Kg/h for 6–12h	1.5–1.9X baseline in 07 days or increase ≥0.3 mg/dL in 48 h	1.5–2.0X baseline or increase ≥0.3 mg/ dL in 48 h	≥1.5X baseline for 7 days for 24 h
**Grade 2**	<0.5 ml/Kg/h for ≥12 h	Increase 2.0–2.9X baseline	>2.0–3.0X baseline	≥2.0X baseline
**Grade 3**	<0.3 ml/Kg/h for ≥24 h or anuria for ≥12 h	≥3.0X baseline or increase ≥4.0 mg/dL or RRT	≥3.0X baseline or increase ≥4.0 mg/dL (w/ increase >0,5 mg/dL) or RRT	≥3.0X baseline or increase ≥4.0 mg/dL (w/ increase >0,5 mg/dL) or RRT

*AKIN = Acute Kidney Injury Network* ^35^ *Cr = creatinine; KDIGO = Kidney Disease Improving Global Outcomes* ^36^ *RIFLE = Risk of renal failure, Injury to the kidney, Failure of kidney function, Loss of kidney function, and End-stage renal failure* ^37^ *RRT = Renal replacement therapy; UO = Urinary Output.*

Additionally, the IVC diameter image may be useful to guide diuretic therapy and thus avoid excessive diuresis beyond the refill rate.^6^

## Evaluation of renal function

The assessment of renal function is challenging, even for nephrologists. The interpretation of laboratory findings may vary between physicians, leading to different medical management pathways. In chronic patients, renal function can be assessed by calculating the estimated glomerular filtration rate (eGFR), which is more accurate in outpatient settings than in intensive care scenarios.^27^ The assessment of tubular integrity is done by quantifying urinary albumin. These two parameters classify the patient according to the stage of kidney disease and the risk of decline in renal function over time.^28^ In ADHF, renal function, as assessed by serum creatinine and electrolytes, should be done at least every 24 hours. The early assessment of diuretic response is assessed by urine output and by urinary sodium measurements. Urinary sodium adds prognostic information^29^ and helps optimize diuretic therapy, with better outcomes^30^ and excellent correlation with 24-hour natriuresis (*r*=0.91 p<0.0001).

In patients with ADHF, assessment of renal function at admission is related to the prognosis during hospitalization. In the ADHERE registry ( ClinicalTrials. gov Identifier: NCT00366639), admissional serum creatinine, blood urea nitrogen, and systolic blood pressure were independent predictors of in-hospital mortality.^31^ Serum creatinine at admission probably reflects the severity of congestion and also identifies, in some cases, the patients with chronic kidney disease. Differently, worsening renal function during the process of decongestion isn't consistently linked to adverse outcomes, since it does not necessarily indicate true kidney injury.^32^ Despite the importance of structural changes in nephron, evidence shows that renal venous congestion plays a more significant role in worsening renal function and diuretic resistance than low renal artery flow, as previously thought.^33,34^ Therefore, adequate treatment of congestion improves renal function. An increase in creatinine during hospitalization may represent true worsening renal function, pseudoworsening renal function or acute kidney injury, and special attention is necessary when the creatinine increases over 100% or above 3.5 mg/dL.^32^ The definition of worsening renal function varies somewhat in the literature but, in general, increases in creatinine greater than 0.3 mg/dL, increases of creatinine beyond 1.5 times the admission value, or an increase beyond 25% and Cr >2.0 mg/dL are the most used in the literature. A summary of different criteria used to diagnose acute kidney damage is shown in *Table 1*. Patients with an increase in serum creatinine who maintain a urinary sodium dosage greater than 50–70 mEq/L on a urine spot collected within two hours of a diuretic dose, a urinary volume of 100–150 mL within 6 hours of diuretic administration, and a total diuresis of 3–4 L within 24 hours^30^ are probably experiencing a false worsening of renal function and, if clear signs of congestion persist, diuretic treatment should be maintained. Of note, this pseudoworsening renal function is not associated with worse outcomes and is probably due to diuresis that exceeds the plasma refill rate, causing transient intravascular ‘hypovolemia’.^19^ In contrast, residual congestion at discharge despite preserved renal function is related to cardiovascular death and hospitalization.^14,20^ gure 2 proposes an algorithm for managing ADHF amidst serum creatinine increases during decongestion.

**Figure 3:**
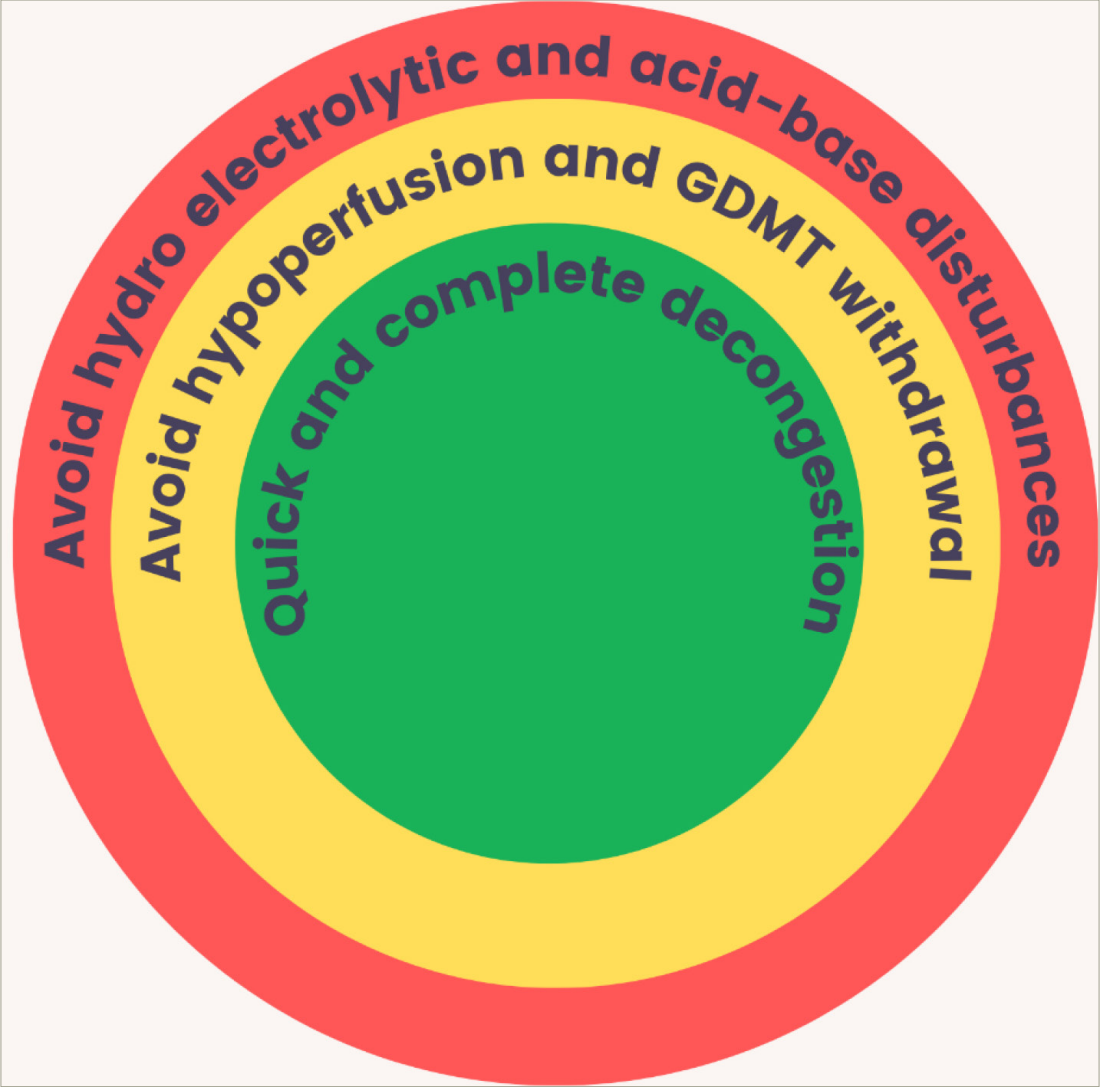
The goals of diuretic therapy in patients with acute decompensated heart failure

## Diuretic therapy in chronic and acute decompensated heart failure

The goals of diuretic therapy are shown in *Figure 3*. The use of diuretics in patients with HF is complex and must be individualized due to the multiple interactions between the neuroendocrine systems. In the era of personalized medicine, new concepts have emerged based on the fact that human physiology is a complex system, subjected to external intervention as well as endogenous adaptive processes.^38^ Clinical judgement may be challenging and the use of biomarkers in this scenario may lead to better outcomes.^39^

Diuretic therapy in ADHF is primarily based on the use of intravenous loop diuretics, under optimal neurohumoral blockade and hemodynamic optimization, to achieve decongestion as soon as possible without harmful consequences.^4^ The use of GDMT, including mineralocorticoid receptor antagonists (MRA), is recommended even in the acute setting.^1^ Therapy with high-dose spironolactone was tested in the ATHENA-HF trial ( ClinicalTrials. gov Identifier: NCT02235077)^40^ in an attempt to obtain greater net fluid loss and natriuresis but the results were neutral as compared to placebo. Therefore, the usual dose of 25 mg of spironolactone is recommended. The use of sodium-g lucose co-transporter 2 inhibitors (SGLT2i) has been shown to promote diuresis without depleting the intravascular space, with a more significant loss of interstitial water and a more sustained natriuresis, opposing the diuretic resistance inherent to loop diuretics.^41–44^ Beta blockers should be maintained in patients with previous use (the dose can be reduced by half) or initiated in naïve patients as soon as euvolemia has been achieved.^1^ Angiotensin-converting enzyme inhibitors, angiotensin receptors blockers or sacubitril/valsartan should be maintained or initiated as soon as possible, provided that no contraindications exist.

In ADHF, furosemide should be used intravenously due to erratic bioavailability by the oral route and reduced absorption by the swollen bowel. Although loop diuretics can cause activation of the RAAS, the final results are positive because early and adequate decongestion is associated with better outcomes.^45^ Response to different strategies in diuretic therapy were assessed in the DOSE-HF Study ( ClinicalTrials. gov Identifier: NCT00577135).^46^ In this trial, low-versus high-dose and intermittent versus continuous infusion were compared. No differences were observed between intermittent doses or continuous infusion regarding clinical improvement or safety at 72 hours , but the ‘intermittent’ group required more dose adjustments at 48 hours and greater accumulated dose of furosemide compared with the ‘continuous infusion’ group. When comparing high and low doses, again, no differences in the rates of symptom improvement and safety were observed, but the high-dose group converted to oral use within 48 hours in a more significant proportion and had faster symptom relief. Of note, the high-dose group had a more significant creatinine increase that was transient and not associated with events within 60 days. In the DOSE-HF trial patients were not evaluated for the presence of diuretic resistance. Therefore, the superiority of one of the strategies in patients with diuretic resistance cannot be fully refuted.^47^ Of note, many patients with ADHF have diuretic resistance or non-adherence to the ambulatory regimen with reports of up to 30% in some registries.^10,12^ Diuretic resistance is challenging and must be recognized early to improve morbidity and decrease mortality.^4,45^

**Figure 4:**
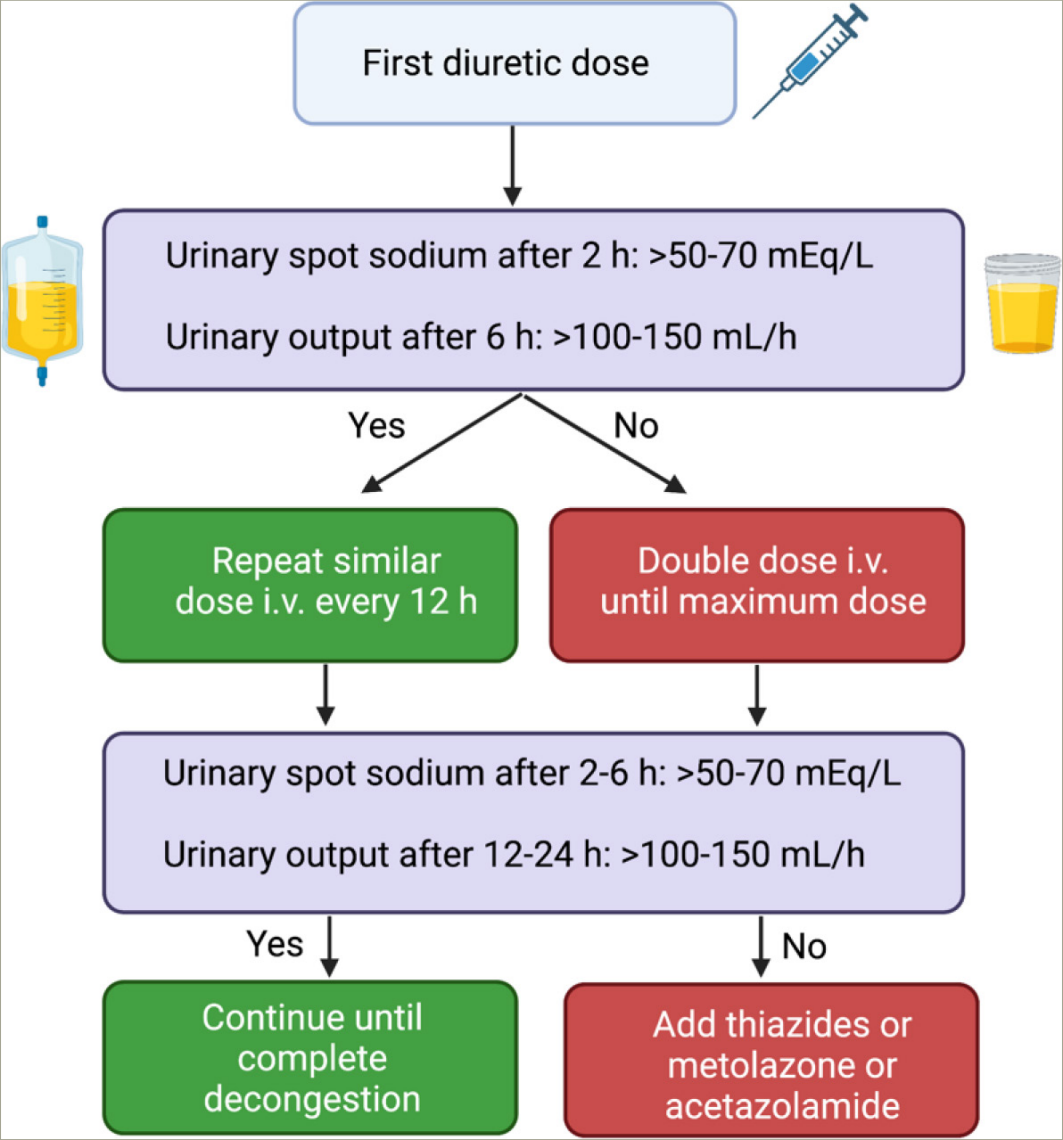
Diuretic therapy in acute heart failure guided by urinary sodium and urinary output

*h = hours*

*Figure created by the authors with* BioRender.com.

**Table 2:** Evaluation of congestion according to the EVEREST score

	EVEREST score	
**Dyspnoea**	0 = None 1 = Seldom	2 = Frequent 3 = Continuous
**Orthopnoea**	0 = None 1 = Seldom	2 = Frequent 3 = Continuous
**Oedema**	0 = None 1 = Seldom	2 = Frequent 3 = Continuous
**Fatigue**	0 = None 1 = Seldom	2 = Frequent 3 = Continuous
**JVD**	0 = ≤6 1 = 6–9	2 = 10–15 3 = ≥15
**Rales**	0 = None 1 = Bases	2 = Up to <50% 3= >50%

Response to diuretic therapy in ADHF can be assessed by measuring urinary output and urinary sodium, which provides early recognition of diuretic resistance and adjustments of doses according to the natriuretic response.^30^ The dose-effect curve of furosemide is sinusoidal, and a minimum amount is required to start the response, which is also limited to a ceiling beyond which no additional response occurs. HF patients need higher doses to achieve the same tubular amount and effect.^7^ As a result, dose increases are more effective than increasing the frequency of administration, especially before adding the second class of diuretics.^33^ An ongoing clinical trial seeks to evaluate diuretic protocols guided by urinary sodium^47^ but, at this time, we suggest using the European Society of Cardiology^4^ (ESC) protocol or the DOSE-HF trial protocol.^33^ In patients already on loop diuretic, the initial dose recommended by the ESC HF guidelines is 1-2 times home daily diuretic dose. In the DOSE-HF trial, a slightly higher dose was used (2.5 times the outpatient dose). The maximum daily dose of furosemide equivalent suggested by the ESC HF guideline is 400-600 mg, for non-naïve diuretic users. For patients not on outpatient diuretic therapy, a single furosemide dose of 20 to 40 mg is indicated (or 40-80 mg, according to the DOSE-HF trial protocol). Urinary sodium levels in a single spot 1-2 hours (h) after diuretic administration <50-70 mEq/L requires a double diuretic dose until the maximum daily amount is achieved.^4,33^ Likewise, urinary output after 6 hours <100-150 mL/h is an indication for double dose as well.^4,33^ The association of a thiazide diuretic after the maximum furosemide dose has been achieved is recommended.^4,33^ Alternatively, acetazolamide in association with furosemide is a valid strategy. In the ADVOR trial ( ClinicalTrials. gov Identifier: NCT03505788),^48^ a randomized parallel-group, double-blind and multicentric study, the use of acetazolamide associated with loop diuretic resulted in more fluid loss, higher natriuresis and higher rates of complete decongestion in three days as compared to placebo. Importantly, there was no difference in WRF, hypokalemia and adverse events between groups.^48^*Figure 4* summarizes the management of diuretic therapy according to the ESC guidelines.

## Achieving euvolemia

It is essential to safely determine euvolemia to switch the route of administration of diuretics, from intravenous to the oral route. However, assessing the volemia may be challenging. Many methods have been proposed for this evaluation. The EVEREST Score (*Table 2*) was set to discharge HF patients safely, quantifying clinical parameters.^13^ A score ≥1 is associated with increasing risk of rehospitalization. Circulating biomarkers may be helpful.^3^ Reduction in natriuretic peptide levels from admission to discharge is indicative of good response and is associated with good prognosis.^3^ Likewise, some laboratory tests that indicate haemoconcentration, such as albumin, haematocrit and haemoglobin have been proposed as surrogate markers for adequate decongestion.^3^ Imaging methods, such as echocardiogram and pulmonary ultrasound, are also useful in the assessment of venous and pulmonary congestion. IVC evaluation and its respiratory variability, B-l ine counts at the lung and the renal venous flow pattern are promising findings to confirm the absence of congestion at bedside.^24,34,49^ Electrical bioimpedance vector analysis is a valuable method to estimate total body water volume and has been proved to be helpful in identifying subclinical congestion.^20^ Finally, measuring liver stiffness as assessed by transient elastography seems to be helpful.^50^ Again, the individual context is crucial for the correct interpretation of volume status, contextualizing signs, symptoms, images and haemodynamic data with comorbidities that may interfere with cardiorenal interactions such as pneumopathies or liver diseases. Once the patient has achieved the euvolemic state, it is mandatory that GMDT be implemented and titrated (if not done before), so that, at hospital discharge, target doses have been reached.

Every HF patient should be discharged in an euvolemic state, after a trial of oral diuretics for at least 24 h before discharge. Some patients may have subclinical congestion at discharge which is difficult to detect. For this reason, patients should be seen early after discharge. A medical visit within 7–15 days after discharge may detect signs of residual congestion and allow adjustments in medications, which may prevent readmissions.^4^

## Conclusion

Interventions tailored to the patient needs are the mainstay of the modern medicine, especially in complex scenarios such as ADHF. The knowledge about congestion has evolved in the last years and has helped to improve treatment. Monitoring natriuresis seems to improve the response to diuretics as it allows for adjustments in diuretic doses. Worsening renal function during the process of decongestion should not preclude the use of diuretics provided that the patient has clear signs of congestion and adequate urine output and/or urinary sodium. Adequate assessment of volemia and the introduction and titration of GMDT are mandatory before discharge. Patients need to be seen as soon as possible after discharge, to assess for residual congestion and thus avoid readmissions.
